# Endoscopic retroperitoneal repair of superior lumbar hernia (Grynfeltt hernia) using self-fixating mesh to prevent nerve injury: a case report

**DOI:** 10.1186/s40792-024-01846-5

**Published:** 2024-03-05

**Authors:** Ryosuke Mizuno, Shusaku Honma

**Affiliations:** Department of Surgery, Kobe City Medical Center West Hospital, 2-4, Ichibancho, Nagataku, Kobe, Hyogo 653-0013 Japan

**Keywords:** Superior lumbar hernia, Laparoscopic retroperitoneal repair, Self-fixating mesh

## Abstract

**Background:**

The primary superior lumbar hernia, also called the Grynfeltt Hernia, is a rare entity; hence, a standard surgical repair method has not been established. It is important for this repair procedure not only to prevent recurrence by placing an adequate size of mesh but also to avoid nerve injury. The endoscopic retroperitoneal approach is considered a useful technique for this procedure, requiring no mobilization of the colon and providing good visibility of the surrounding nerves. A self-fixating mesh does not require a traumatic fixation, which is considered advantageous for nerve preservation.

**Case presentation:**

A 72-year-old woman, complaining of left lumbar swelling, was diagnosed with a primary left superior lumbar hernia and underwent endoscopic retroperitoneal hernia repair. With the patient in the right lateral decubitus position, five small ports were inserted, and the retroperitoneal space was dissected. The 2.0 × 1.0-cm hernial orifice was found, and the 12th subcostal nerve above the hernial orifice and the iliohypogastric and the ilioinguinal nerves below the hernial orifice were identified. A 15 × 10-cm self-fixating mesh was placed in the retroperitoneal space without mesh tacking. The postoperative course was uneventful, and the patient was discharged on the first postoperative day. Two years after surgery, there was no sign of recurrence, and chronic pain and neuropathic symptoms were absent.

**Conclusions:**

Endoscopic retroperitoneal repair of primary superior lumbar hernia using a self-fixating mesh seems to be useful for nerve preservation.

## Background

Primary superior lumbar hernias, first described by Grynfeltt in 1866 and thus called the Grynfeltt hernia, is an uncommon disease [1]. The lumbar region has two vulnerable anatomical sites: the upper and lower triangles. The upper vulnerable site, called the triangle of Grynfeltt, is where superior lumbar hernias arise. The triangle of Grynfeltt is an inverted triangle with the medial border being the quadratus lumborum muscle or sacrospinalis muscle; the lateral border, the internal oblique muscle; and the superior border, the 12th rib [1]. The triangle is surrounded by many nerves, including the 12th subcostal, iliohypogastric, ilioinguinal, and lateral femoral cutaneous nerves. Therefore, the repair of superior lumbar hernias is associated with a risk of nerve injury. Although several surgical approaches for superior lumbar hernias have been reported, there is no standardized surgical method for this hernia due to its relative rarity. The endoscopic retroperitoneal approach is a minimally invasive surgery for the superior lumbar hernia, requiring no mobilization of the colon or other intraperitoneal organs. It has the advantages of minimal and sufficient dissection and excellent visibility of the surrounding nerves. In this report, we present a case of a primary superior lumbar hernia that was successfully repaired via the endoscopic retroperitoneal approach using a self-fixating mesh without nerve injury.

## Case presentation

**Fig. 1:**
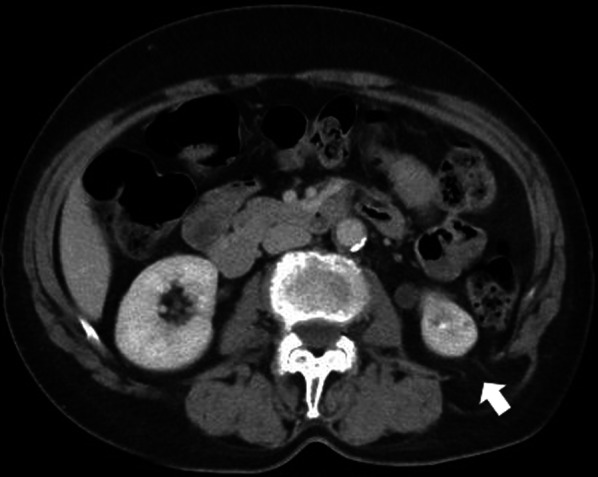
Abdominal computed tomography. Abdominal computed tomography revealed the left superior lumber hernia (arrow). The size of the fascial defect was 2.0 × 1.0 cm

A 72-year-old woman presented at our hospital with a complaint of a reducible lump in her left lumbar region. She had no previous history of abdominal surgery but had undergone right breast cancer surgery 9 years previously. She had a medical history of dermatomyositis treated with topical steroids. Physical examination revealed a mild reducible bulge in the left lumbar region during standing forward bending, and computed tomography showed a fascial defect in the left lumbar region through which retroperitoneal fat protruded (Fig. 1). The size of the fascial defect was 2.0 × 1.0 cm. With a diagnosis of primary superior lumbar hernia, endoscopic retroperitoneal hernia repair was performed.

**Fig. 2:**
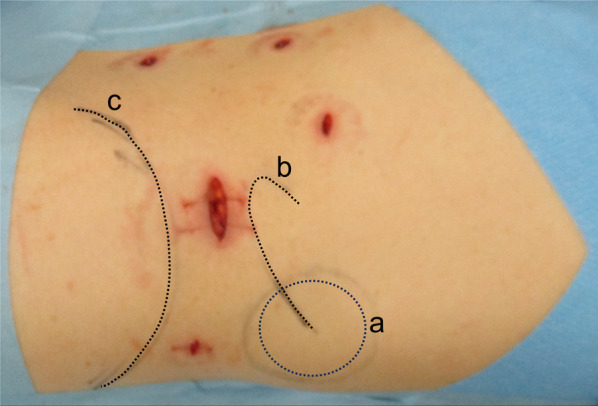
Schema of endoscopic ports placement. Patient was placed in the right lateral decubitus position. A 2-cm skin incision was made 2 cm caudal to the 11th rib. **a** Superior lumbar hernia bulge visible from the body surface. **b** 11th rib. **c** Iliac crest

**Fig. 3:**
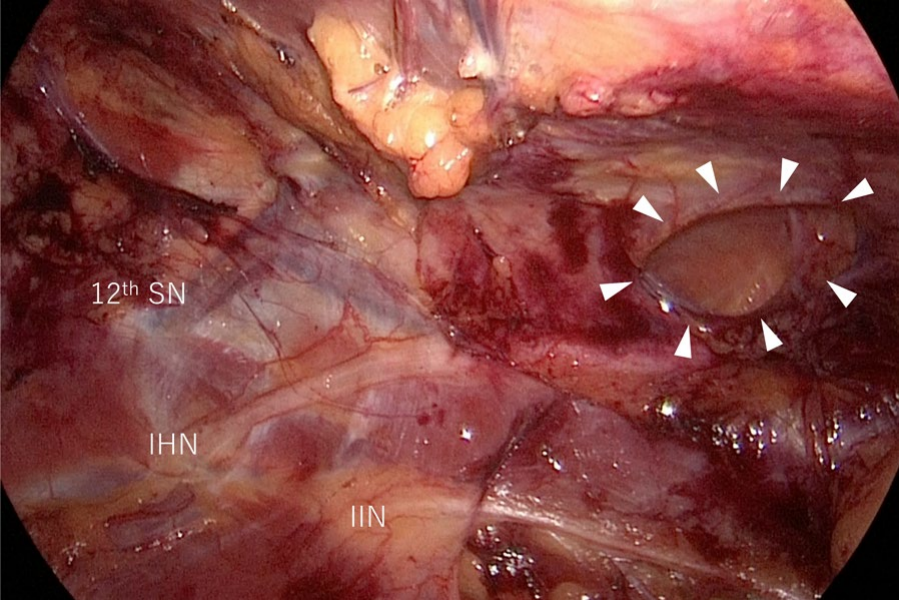
Intraoperative view after dissection of the retroperitoneal space. Intraoperative view indicates a hernial orifice (arrowheads). 12th SN: 12th subcostal nerve; IHN: iliohypogastric nerve; IIN: ilioinguinal nerve

**Fig. 4:**
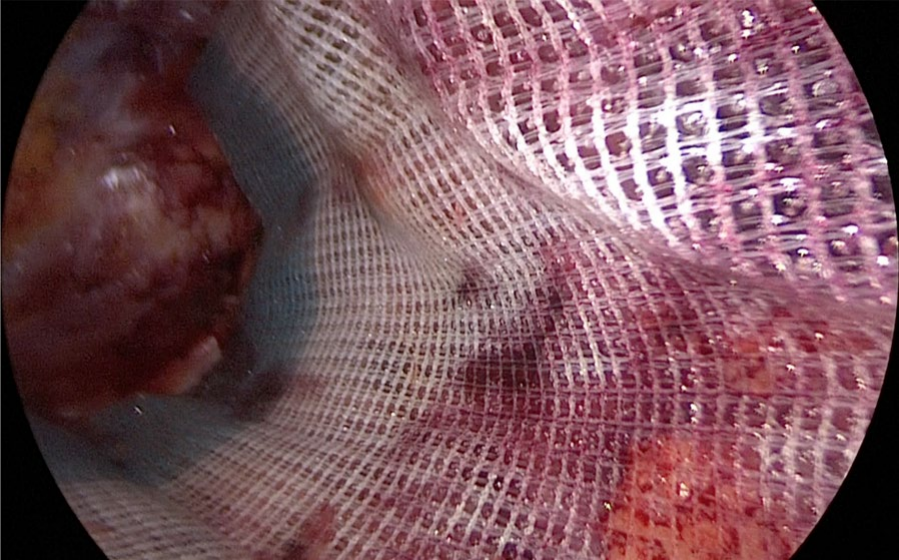
Intraoperative view after mesh placement. A self-fixating mesh of 15 × 10 cm was placed without traumatic mesh fixation

The patient was placed in the right lateral decubitus position under general anesthesia. A 2-cm skin incision was made 2 cm caudal to the 11th rib (Fig. 2); the external oblique, internal oblique, and transversus abdominis muscles were split using the open technique; and finally, the transverse fascia was incised to reach the retroperitoneal space. After some blunt dissection using the surgeon’s fingers, a balloon trocar was inserted, and the retroperitoneal space was widened via laparoscopic blunt balloon dissection. A total of four 5-mm trocars and one 12-mm trocar were inserted into the left lateral abdomen, and dissection of the retroperitoneal space was continued. The iliohypogastric and ilioinguinal nerves, which run over the quadratus lumborum muscle, were identified and preserved (Fig. 3). The hernial orifice was located lateral to the quadratus lumborum muscle, just caudal to the 12th rib, and measured approximately 2.0 × 1.0 cm (Fig. 3). A self-fixating mesh of 15 × 10 cm (ProGrip™ Medtronic, Dublin, Ireland) was placed without traumatic mesh fixation (Fig. 4). The operative time was 144 min, and blood loss was minimal.

**Fig. 5:**
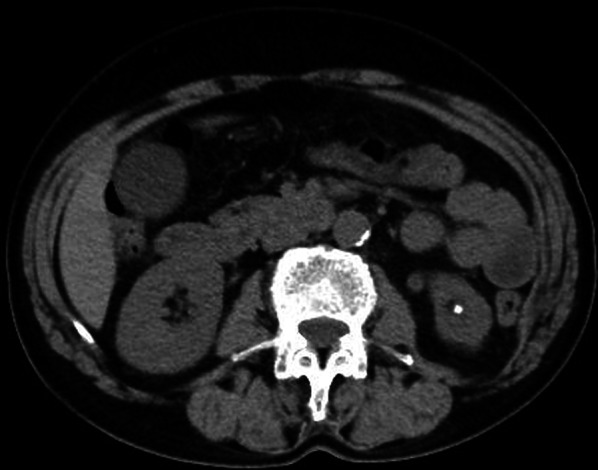
Postoperative abdominal computed tomography. Two years after the surgery, there was no sign of recurrence

The postoperative course was uneventful, and the patient was discharged the next day. Two years after the surgery, there was no sign of recurrence (Fig. 5), and no chronic pain or neuropathic symptoms were observed.

## Discussion

Superior lumber hernias arise from the triangle of Grynfeltt, an inverted triangle with the medial border being the quadratus lumborum or sacrospinalis muscle; the lateral border, the internal oblique muscle; and the superior border, the 12th rib [1]. The floor of this triangle is the transversus abdominis aponeurosis and the transverse fascia, and there is a vulnerable area just below the ribs that is not covered by the external oblique muscle.

Primary superior lumbar hernias are uncommon; hence, there is no standard surgical method to treat this condition. From the early 1900s, primary suturing of the hernial orifice via the open anterior approach and augmentation with a muscle flap have been performed. Due to the high recurrence rate of primary suturing [2], tension-free hernia repair using a surgical mesh has been preferred in recent years. In 1950, Thorek was the first to report a mesh repair of a superior lumbar hernia [3]. With the spread of minimally invasive surgery, the laparoscopic transabdominal preperitoneal approach for traumatic superior lumbar hernia was reported in 1996 [4], and the endoscopic retroperitoneal approach for incisional superior lumbar hernia was reported in 1999 [5]. For the primary superior lumbar hernia, Heniford was the first to report a laparoscopic transabdominal approach with an intraperitoneal onlay mesh technique in 1997 [6]. A retrospective cohort study comparing the laparoscopic transabdominal preperitoneal and open anterior approaches for lumbar hernias found that there was no significant difference in complication and recurrence rates, whereas fewer incidences of postoperative pain and shorter hospital stay were observed in the laparoscopic group than those in the open group [7]. To our knowledge, there have been no studies directly comparing transabdominal and retroperitoneal approaches in laparoscopic surgery for superior lumbar hernias; nevertheless, the transabdominal approach reportedly requires mobilization of the colon to reach the triangle of Grynfeltt, while the retroperitoneal approach does not require such mobilization and allows an easier dissection, resulting in a reduced operative time [8, 9]. In the present case, the retroperitoneal approach was selected considering the aforementioned points as advantages.

On the other hand, a disadvantage of the retroperitoneal approach for hernia repair is the limited surgical field and the difficulty in manipulating forceps, which can result in insufficient dissection and concerns about recurrence. In this case, we started with the usual 3-port hernia repair and adapted to the situation by adding two 5-mm trocars, achieving safe and adequate dissection. We believe that the aesthetic disadvantages and the risk of incisional hernia are minimal with a 5-mm incision [10].

When repairing the superior lumbar hernia, careful procedures are required to avoid intraoperative nerve injury because many nerves travel around the triangle of Grynfeltt, including the subcostal, iliohypogastric, ilioinguinal, and lateral femoral cutaneous nerves, all of which must be preserved [1]. In laparoscopic superior lumbar hernia repair, both peritoneum and retroperitoneal fat should be dissected from the lumbar wall. The risk of nerve injury is higher in such dissection because the surface of the critical nerves is not protected by the fatty tissue [11]. The most significant cause of nerve injury is considered to be traumatic mesh fixation using a tacker. Self-fixating mesh, which can eliminate traumatic mesh fixation, is effective for the preservation of nerves and decrease of chronic pain [12, 13]. As shown in Table 1, there were 7 previous reports and 17 cases of primary superior lumbar hernia repaired through the endoscopic retroperitoneal approach [8, 14–19]. Among these 17 cases, only 3 required traumatic fixation, likely due to the closed retroperitoneal space characteristic of the retroperitoneal approach. There were only 2 cases that used self-fixating mesh without traumatic fixation, and neither showed any short-term postoperative neuropathic symptoms. However, the follow-up period was brief, leaving the outcomes regarding long-term chronic pain unclear [16, 17]. In the present case, a long-term postoperative follow-up of 2 years was conducted, with no chronic pain or recurrence observed. We believe that the endoscopic retroperitoneal approach and self-fixating mesh are the good combination for superior lumbar hernia repair, and that it can be performed safely by general surgeons if they are aware of the characteristic retroperitoneal neuroanatomy. To validate the effectiveness of this surgical method for superior lumbar hernias, prospective studies will be necessary.

**Table 1:** Cases of primary superior lumbar hernia repaired through the endoscopic retroperitoneal approach

No.	Author	Year	Age	Sex	Operative time (minutes)	Postoperative hospital stay (days)	Mesh	Fixation	Follow-up duration (months)	Recurrence	Chronic pain
1	Habib et al. [8]	2003	65	M	ND	2	Polypropylene mesh	Traumatic fixation	24	None	ND
2	Lim et al. [14]	2011	76	F	ND	5	Polypropylene mesh	Traumatic fixation	11	None	ND
3	Wei et al. [15]	2014	76	F	ND	4	Polypropylene mesh	Traumatic fixation	18	None	ND
4	Li et al. [16]	2019	62	F	ND	1	Self-fixating mesh	None	2	None	ND
5	Nakahara et al.[17]	2020	65	F	50	5	Self-fixating mesh	None	9	None	ND
6	Li et al. [18]	2021	56	M	55	2	Polypropylene mesh	Chemical glue	15	None	None
7		2021	70	F	50	2	Polypropylene mesh	chemical glue	14	None	None
8		2021	68	F	45	2	Polypropylene mesh	Chemical glue	9	None	None
9		2021	54	F	50	1	Polypropylene mesh	Chemical glue	9	None	None
10		2021	69	F	45	1	Polypropylene mesh	Chemical glue	8	None	None
11		2021	67	F	45	1	Polypropylene mesh	Chemical glue	7	None	None
12		2021	66	F	40	1	Polypropylene mesh	Chemical glue	5	None	None
13		2021	74	M	50	1	Polypropylene mesh	Chemical glue	3	None	None
14		2021	55	F	60	1	Polypropylene mesh	Chemical glue	3	None	None
15		2021	75	F	50	2	Polypropylene mesh	Chemical glue	2	None	None
16	Li et al. [19]	2023	70	F	66	2	Polypropylene mesh	None	24	None	None
17		2023	51	F	60	3	Polypropylene mesh	None	10	None	None
18	Our case		72	F	144	1	Self-fixating mesh	None	24	None	None

*ND* not described

## Conclusions

We experienced a case of a primary superior lumbar hernia successfully repaired via the endoscopic retroperitoneal approach with a self-fixating mesh. This surgical method may be useful for nerve preservation, and can be performed safely by general surgeons.

## Data Availability

The datasets supporting the conclusions of this article are included in this paper.
